# Haploinsufficiency as a disease mechanism in *GNB1*‐associated neurodevelopmental disorder

**DOI:** 10.1002/mgg3.1477

**Published:** 2020-09-12

**Authors:** Laura Schultz‐Rogers, Ikuo Masuho, Filippo Pinto e Vairo, Christopher T. Schmitz, Tanya L. Schwab, Karl J. Clark, Lauren Gunderson, Pavel N. Pichurin, Klaas Wierenga, Kirill A. Martemyanov, Eric W. Klee

**Affiliations:** ^1^ Center for Individualized Medicine Mayo Clinic Rochester MN USA; ^2^ Department of Neuroscience The Scripps Research Institute Jupiter FL USA; ^3^ Department of Clinical Genomics Mayo Clinic Rochester MN USA; ^4^ Department of Biochemistry and Molecular Biology Mayo Clinic Rochester MN USA; ^5^ Department of Medical Genetics Mayo Clinic Jacksonville FL USA

## Abstract

**Background:**

*GNB1* encodes a subunit of a heterotrimeric G‐protein complex that transduces intracellular signaling cascades. Disruptions to the gene have previously been shown to be embryonic lethal in knockout mice and to cause complex neurodevelopmental disorders in humans. To date, the majority of variants associated with disease in humans have been missense variants in exons 5‐7.

**Methods:**

Genetic sequencing was performed on two patients presenting with complex neurological phenotypes including intellectual disability, hypotonia, and in one patient seizures. Reported variants were assessed using RNA sequencing and functional BRET/BiFC assays.

**Results:**

A splice variant reported in patient 1 was confirmed to cause usage of a cryptic splice site leading to a truncated protein product. Patient 2 was reported to have a truncating variant. BRET and BiFC assays of both patient variants confirmed both were deficient in inducing GPCR‐induced G protein activation due to lack of dimer formation with the Gγ subunit.

**Conclusion:**

Here, we report two patients with functionally confirmed loss of function variants in *GNB1* and neurodevelopmental phenotypes including intellectual disability, hypotonia, and seizures in one patient. These results suggest haploinsufficiency of *GNB1* is a mechanism for neurodevelopmental disorders in humans.

## Graphical abstract

Missense variants in GNB1 are associated with a complex neurodevelopmental disorder. Here we present two additional patients with overlapping phenotypes including developmental delay, hypotonia, intellectual disability, and seizures in one patient. Both patients have presumed loss‐of‐function variants which we have shown functionally to cause loss of protein function, thus adding evidence that haploinsufficiency is a disease mechanism associated with GNB1.

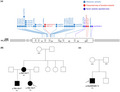

## 1 INTRODUCTION

The gene *GNB1* (guanine nucleotide binding protein (G protein), beta polypeptide 1; OMIM: 616973 and OMIM:613065) encodes the Gβ subunit of a heterotrimeric G‐protein complex which additionally includes Gα and Gγ subunits. This complex functions to transduce a variety of intracellular signaling cascades (Ford et al., 1998). Homozygous loss of *Gnb1* in mice is embryonically lethal with affected embryos showing abnormal brain morphology and defects in neural precursor differentiation and proliferation (Okae & Iwakura, 2010). In humans, variants in this gene have been associated with a neurodevelopmental disorder (NDD; OMIM: 616973, Mental retardation, autosomal dominant 42) with patients presenting with global developmental delay, hypotonia, and epilepsy (Brett et al., 2017; Endo et al., 2020; Hemati et al., 2018; Jones et al., 2019; Lohmann et al., 2017; Peng et al., 2019; Petrovski et al., 2016; Steinrucke et al., 2016; Szczaluba et al., 2018). Other prevalent phenotypes reported include brain abnormalities, ophthalmological disorders, growth restriction, movement disorders, and non‐specific dysmorphisms (Revah‐Politi, Sands, Colombo, Goldstein, & Anyane‐Yeboa, 1993). Some reported phenotypes show variable expressivity. For example, of the 31 patients presented in Petrovski et al. and Hemati et al., only 16 patients (51.6%) were reported with epilepsy/seizures, and similarly there were 16/31 patients with reported brain abnormalities on magnetic resonance imaging (MRI). Overall, the phenotypes are consistent with a severe neurodevelopmental disorder leading to global developmental delay with many patients never obtaining the ability to ambulate or communicate verbally.

**FIGURE 1:**
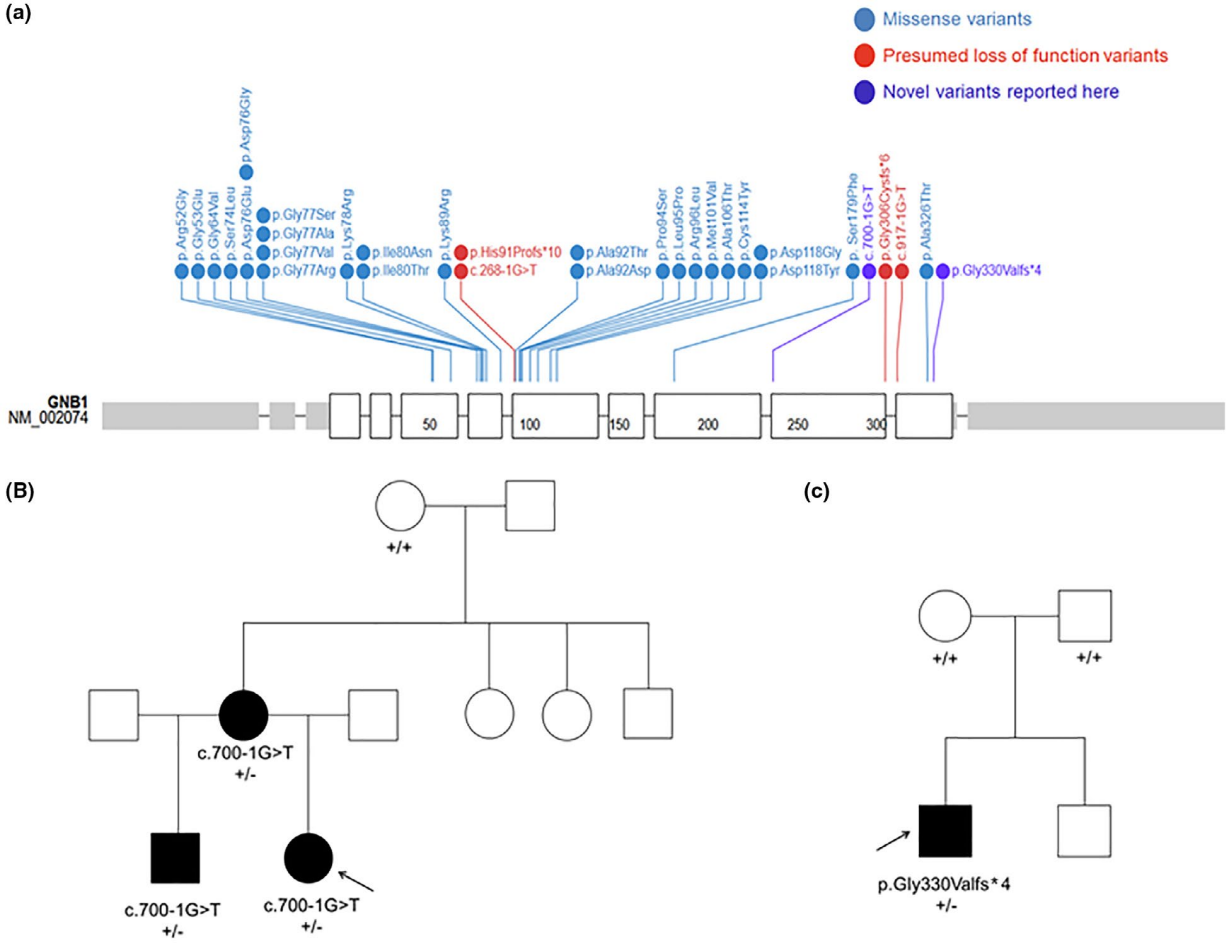
Variants in GNB1 currently reported in the literature as associated with NDD. (a) Thirty variants are currently associated with neurodevelopmental disorder (NDD) in the literature. Twenty‐six are missense variants (blue) while only four presumed loss of function (LoF) variants (red) have been previously reported. The variants presented in this report are also presumed LoF (purple). Figure generated using https://prote​inpai​nt.stjude.org/. (b) Pedigree for Patient 1. Individuals who were sequenced are indicated with genotype, affected individuals are in black. (c) Pedigree for Patient 2. Sequencing was performed on the parent‐child trio with genotypes indicated and affected individuals in black. *GNB1* GenBank reference sequence NM_002074.4.

The vast majority of variants that have been associated with NDD in *GNB1* are missense variants clustering in exons 5‐7 (26/30 reported variants; Figure 1a). This region is critical for the heterotrimeric interaction between Gα/Gβγ within the G protein complex (Clapham & Neer, 1997; Graf et al., 1992; Lambright et al., 1996; Oldham & Hamm, 2008; Wall et al., 1995). Functional studies have been performed investigating the effect of several different missense variants on the ability of Gβ to properly interact with Gα and Gγ and transduce signals initiated by the D1 dopamine receptor (D1R; Lohmann et al., 2017). These studies demonstrated that specific missense variants (p.Arg52Gly, p.Gly64Val, p.Ala92 Thr, p.Pro94Ser, p.Arg96Leu, p.Ala106 Thr, and p.Asp118Gly) lead to LoF of the G‐protein complex through deficits in Gβγ dimer formation, heterotrimer assembly, or receptor‐driven G protein activation. Of note, several cohort studies have not found any genotype‐phenotype association between the severity of phenotype and location of the reported missense variant (Hemati et al., 2018; Lohmann et al., 2017; Petrovski et al., 2016).

Of the 52 patients currently reported in the literature with *GNB1*‐associated NDD, 48 are reported to have *de novo* variants. One patient is reported with unknown inheritance due to lack of parental samples, and three patients are reported with inherited variants (Lohmann et al., 2017). The three inherited cases are all in apparently unrelated families but interestingly all individuals have the same c.287G>T, p.Arg96Leu missense variant. Whether or not the heterozygous parents in these cases are affected and to what degree and whether or not mosaicism was tested for in the parents was not disclosed. Additionally, gene intolerance metrics reported by the gnomAD database confirm *GNB1* is highly intolerant to missense variation with a Z score of 3.83. Interestingly, the gene is also highly intolerant to loss‐of‐function (LoF) variation with a pLI of 1 and zero observed single nucleotide LoF variants reported in gnomAD. Altogether, this data indicate that a single damaging variant in *GNB1* can lead to disease, and in some cases this can result from autosomal dominant inheritance from a mildly affected parent possibly due to mosaicism.

Despite the fact that *GNB1* is highly intolerant to LoF variation, there is relatively limited evidence associating these variants with disease. Only four patients have been reported with presumed LoF truncating or splice variants, and these variants have not been functionally tested. Here, we report two additional individuals presenting with developmental delay, intellectual disability, hypotonia, and presumed LoF variants in *GNB1*. We performed functional testing using a reporter system that can assess GPCR‐induced G protein activation and subunit interaction which demonstrated the patient variants lack normal protein function. Together, these results add further evidence that haploinsufficiency is a disease mechanism associated with *GNB1*.

### 1.1 Case reports

Patient 1 was born at term with pregnancy complicated by lack of prenatal care, and tobacco use. She had delayed speech with about 12 word vocabulary by 30 months of age. Her motor developmental milestones were essentially normal, although some records have indicated delayed motor skills. Mild hypotonia in childhood was reported by a neurologist on one occasion, but did not seem to be noted subsequently. From medical records available to us, her weight was normal until about age 3years when she started having obsession with food and food craving. She developed obesity, apparently not amenable to dietary and exercise interventions. Molecular analysis for Prader‐Willi syndrome was negative. The patient was diagnosed with mild intellectual disability, depressive disorder, ADHD, and violent behavior. An MRI of the brain was normal at 9 years old. At age 13, the patient was found to have nocturnal episodes with EEG correlate and was diagnosed with frontal lobe seizures. Fragile X molecular analysis, chromosomal microarray, metabolic screening testing were all negative/normal. The patient is currently 18 years old and continues to display behavioral dyscontrol with multiple episodes of suicidal ideation. The patient's family history is significant for reported history of father with moderate mental retardation, mother with mild/moderate mental retardation and obesity, and a maternal half‐brother with seizures, ADHD, and speech delay (Figure 1b).

Patient 2 was born at term with an unremarkable prenatal history. Hypotonia was noted a few weeks after birth, and developmental milestones were delayed with walking at 3 years of age and speaking at 4 years of age. The patient was diagnosed with autism at 3 years old and was evaluated for suspected Prader‐Wili and Fragile X syndromes due to his developmental delays and obesity, though testing for both was negative. Array CGH revealed a maternally inherited duplication (arr[GRCh37]3p26.3(1038431_1428517)x3) that was thought to be benign. The patient is currently 40 years old and presents with intellectual disability, episodes of psychiatric illness, obesity, and Crohn's disease. The family history is negative for other similarly affected individuals (Figure 1c).

## 2 Methods

### 2.1 Ethical compliance

This study was approved by the Mayo Clinic institutional review board and all participants provided written informed consent for genetic testing.

### 2.2 Genetic sequencing

Whole exome sequencing for patient 1 was performed by GeneDx. The mean depth of coverage was 97x and the quality threshold (percentage of sequence covered by at least 10 reads) was 98.7%. A proprietary capture system was used for next‐generation sequencing and targets were sequenced with paired‐end reads on an Illumina platform. Reads were aligned to GRCH37/UCSC hg19. Sequence variants and deletions or duplications involving three or more coding exons were called with XomeAnalyzer. Variants were reported according to the Human Genome Variation Society (HGVS) guidelines. Reported variants were confirmed by an appropriate orthogonal method. Sequencing for patient 2 was also performed by GeneDx as per above, however, as part of the Autism/ID Xpanded Panel analysis was targeted to the 2300+ genes included in the panel.

### 2.3 RNA sequencing

RNA sequencing was performed on whole blood samples as previously described (Oliver et al., 2019).

### 2.4 cDNA constructs

Human dopamine D2 receptor in pcDNA3.1(+) was purchased from the cDNA Resource Center and was constructed by GenScript. Plasmid encoding Venus 156‐239‐Gβ1 and Venus 1‐155‐Gγ2 were a gift from N. Lambert (Georgia Regents University; Hollins, Kuravi, Digby, & Lambert, 2009). masGRK3ct‐Nluc‐HA were previously described (Gulati et al., 2018). Human GαoA in pcDNA3.1(+) and Venus 156‐239‐Gβ1 mutants in pcDNA3.1(+) were synthesized by GenScript.

Two selected variants in *GNB1* (NM_002074.4) were introduced via site‐directed mutagenesis in the plasmid Venus 156‐293 Gβ1 pcDNA3.1(+). A variant sequence‐introducing primer was paired with a common insert primer to produce two insert fragments. Each fragment was PCR amplified (Platinum^TM^ *Taq* DNA Polymerase High Fidelity, Thermo Fisher 11304011) and gel purified (QIAEX II Gel Extraction Kit, QIAGEN 20021). The PCR‐amplified fragments were sub‐cloned into the linearized plasmid cut by KpnI‐HF (NEB R3142) and BbsI‐HF (NEB R3539) using the Gibson Assembly Cloning Kit (NEB E2611S). The integrity of all constructs was verified through Sanger DNA sequencing. See Table 1 for primer sequences.

**TABLE 1:** Primers and sequences.

Variant primer	Sequence
Common insert	
Forward (F)	CTAGCGTTTAAACTTAAGCTTGGTAC
Reverse (R)	CGCATCCCCAGCATGCCT
p.Gly330Valfs*4	
F	ATGGCTGTGGCGACGGTCCTGGGATAGC
R	TATCCCAGGACCGTCGCCACAGCCATGC
p.Phe234 Thrfs*16	
F	ATCAATGCCATTTGCACGACGCCACCTGCA
R	CAGGTGGCGTCGTGCAAATGGCATTGATGT

*GNB1* GenBank reference sequence NM_002074.4.

### 2.5 Antibodies

Anti‐Gβ (06‐238) were purchased from MilliporeSigma. HRP‐conjugated anti‐mouse antibody (115‐035‐174) was purchased from Jackson ImmunoResearch.

### 2.6 Real‐time monitoring of G protein activity by fast kinetic BRET assay

BRET experiments were performed as previously reported with slight modifications (Masuho, Martemyanov, & Lambert, 2015; Masuho, Ostrovskaya, et al., 2015). Briefly, 293T/17 cells were grown in DMEM supplemented with 10% FBS, minimum Eagle's medium non‐essential amino acids, 1 mM sodium pyruvate and antibiotics (100 units/ml of penicillin and 100 mg/ml of streptomycin) at 37°C in a humidified incubator containing 5% CO_2_. Cells were transfected with Lipofectamine LTX transfection reagents (6 µl/3.5‐cm dish) and PLUS (5 µl/3.5‐cm dish). Dopamine D2 receptor, GαoA, Venus‐156‐239‐Gβ1, Venus‐1‐155‐Gγ2, and masGRK3ct‐Nluc‐HA (total 5 µg) were used at a 1:2:1:1:1 ratio (ratio 1 = 0.21 µg of plasmid DNA). BRET and Venus intensity measurements were performed using a microplate reader (POLARstar Omega, BMG Labtech) equipped with two emission photomultiplier tubes. All measurements were performed at room temperature. The BRET signal is determined by calculating the ratio of the light emitted by Venus‐Gβ1γ2 (535 nm) over the light emitted by masGRK3ct‐Nluc‐HA (475 nm). The average baseline value recorded before agonist stimulation was subtracted from BRET signal values, and the resulting difference (∆BRET ratio) was plotted as traces.

### 2.7 Western blotting

For each 3.5‐cm dish, transfected cells were lysed in 1 ml of sample buffer (62.5 mM tris–HCl, pH 6.8, 2 M urea, 2% SDS, 5% 2‐mercaptoethanol, 10% glycerol, bromophenol blue [0.08 mg/ml]). Western blotting analysis of proteins was performed after samples were resolved by SDS–polyacrylamide gel electrophoresis and transferred onto PVDF membranes. Blots were blocked with 5% skim milk in PBS containing 0.1% Tween 20 (PBST) for 30 min at room temperature, which was followed by a 90‐min incubation with anti‐Gβ antibody (1:1,000) diluted in PBST containing 1% skim milk. Blots were washed in PBST and incubated for 45 min with a 1:10,000 dilution of secondary antibodies conjugated with horseradish peroxidase (HRP) in PBST containing 1% skim milk. Western blotting was performed with BlotCycler automated western blot processor (Precision Biosystems). Proteins were visualized with Kwik Quant imager (Kindle Biosciences).

## 3 RESULTS

Both patients presented to the Department of Clinical Genomics at the Mayo Clinic for further evaluation. Clinical whole exome sequencing was ordered for patient 1. A maternally inherited likely pathogenic NM_002074.4(*GNB1*):c.700‐1G>T:IVS9‐1G>T splice variant in intron 9 was reported. The patient also has a truncating NM_003128.2(*SPTBN1*):c.5361G>A, p.Trp1781* variant reported in the gene of uncertain significance *SPTBN1*. The inheritance for this variant, while not inherited maternally, could not be fully determined as a paternal sample was not provided for testing. *SPTBN1* is not currently associated with any disease in OMIM and its relevance to the patient phenotype is still under investigation though it could also be a contributing factor. The c.700‐1G>T splice variant in *GNB1* is not reported in the gnomAD database and occurs at the canonical splice acceptor site upstream of exon 10. The *in silico* prediction tool SpliceAI (Jaganathan et al., 2019) predicts complete ablation of this acceptor site (score = 0.9989) and predicts the use of a novel acceptor site 38 base pairs into exon 10 (score = 0.9764). Segregation testing was performed and the patient's affected brother was found to be heterozygous for the variant while the unaffected maternal grandmother did not harbor the variant. Thus, the c.700‐1G>T splice variant in *GNB1* appears to segregate in affected family members in an autosomal dominant manner (Figure 1b).

For patient 2 an Autism/ID Xpanded panel was ordered and a *de novo* heterozygous NM_002074.4(*GNB1*):c.987_988delAG, p.Gly330Valfs*4 frameshift variant of uncertain significance was reported. This variant is not reported in gnomAD. The frameshift variant is predicted to cause a premature stop codon in the last exon, eight amino acids upstream of the canonical stop codon. As such it is unlikely that this transcript will undergo nonsense‐mediated decay.

**FIGURE 2:**
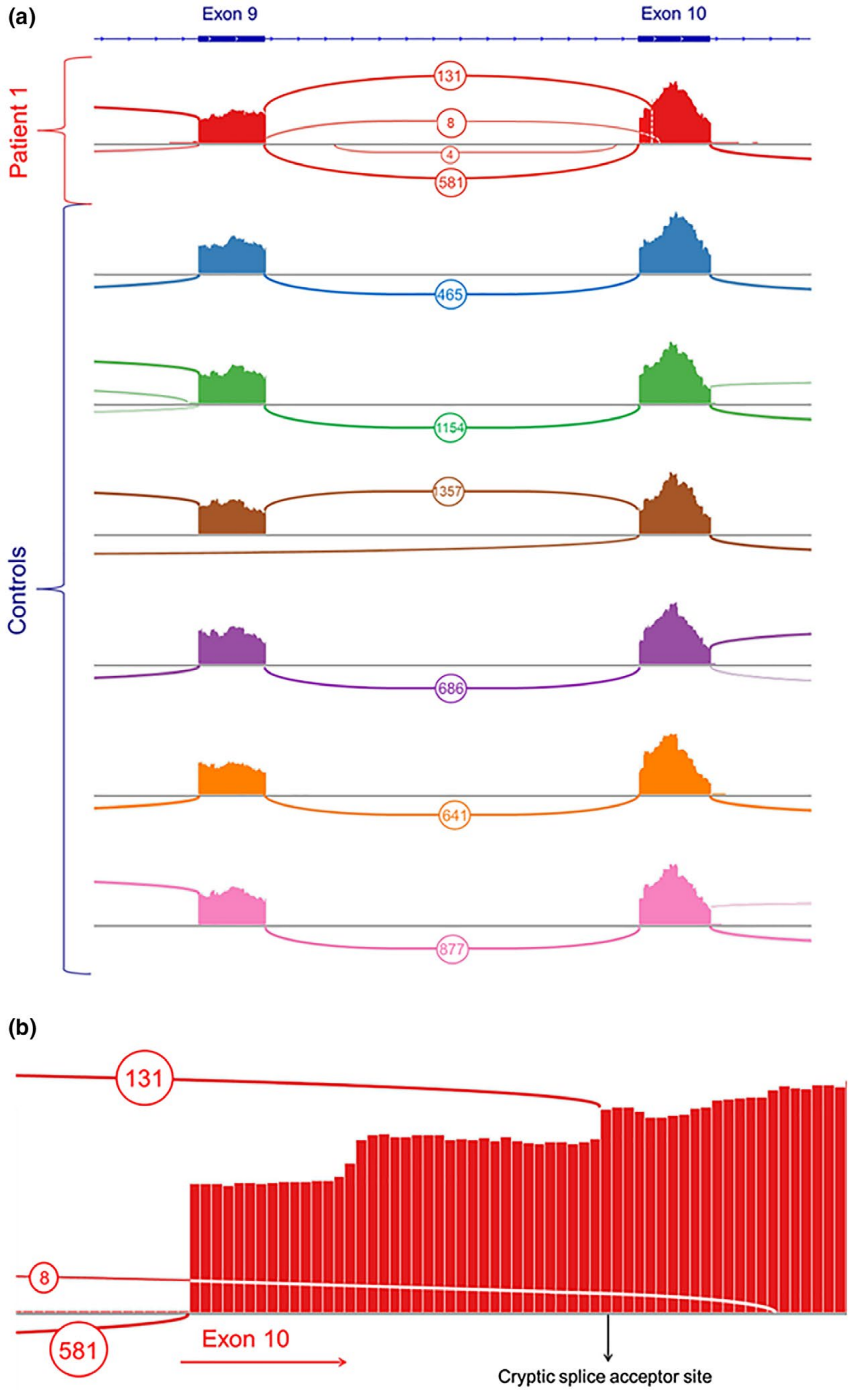
RNA sequencing reveals aberrant splicing in GNB1 in Patient 1. (a) Splicing reads from RNA sequencing of whole blood are shown for Patient 1 as well as 6 unrelated, unaffected control patients. No reads skipping exon 10 were detected in the patient, and none of the control samples displayed splicing into alternate cryptic splice sites in exon 10. (b) Aberrant splicing into exon 10 in Patient 1 includes 131 reads splicing 38 base pairs into exon 10 and 8 reads splicing 54 base pairs into exon 10. About 581 reads represent normal splicing from exon 9 to exon 10. *GNB1* GenBank reference sequence NM_002074.4.

We performed RNA sequencing on whole blood from patient 1 to confirm the aberrant splice predictions. RNA sequencing results from six unrelated cases with no reported variants in *GNB1* demonstrate the average number of reads splicing from exon 9 to exon 10 is 863, with a range from 465 reads to 1357 reads (Figure 2a). Patient 1 has a total of 720 reads splicing from exon 9 to exon 10. However, 131 of those reads (18%) map from exon 9 to the predicted alternate splice site 38 base pairs into exon 10 (Figure 2b). An additional eight reads spliced from exon 9 to an alternate splice site 54 base pairs into exon 10. No reads splicing from exon 9 to further downstream exons were reported suggesting the c.700‐1G>T splice variant in patient 1 leads to the use of a cryptic splice acceptor site rather than skipping of exon 10. Notably, there was no observed usage of this cryptic splice site in any of the six unrelated controls. The functional outcome of this aberrant splicing is an out of frame transcript beginning at amino acid position 234 with a novel stop codon leading to premature truncation at amino acid position 250 (p.Phe234Thrfs*16). Because we only observe 18% of alternatively spliced transcript, it is possible that some of the damaged transcript is undergoing nonsense‐mediated decay (NMD), however, it cannot be ruled out that the remaining transcript from the maternal allele is actually undergoing normal splicing to produce a wild‐type transcript.

**FIGURE 3:**
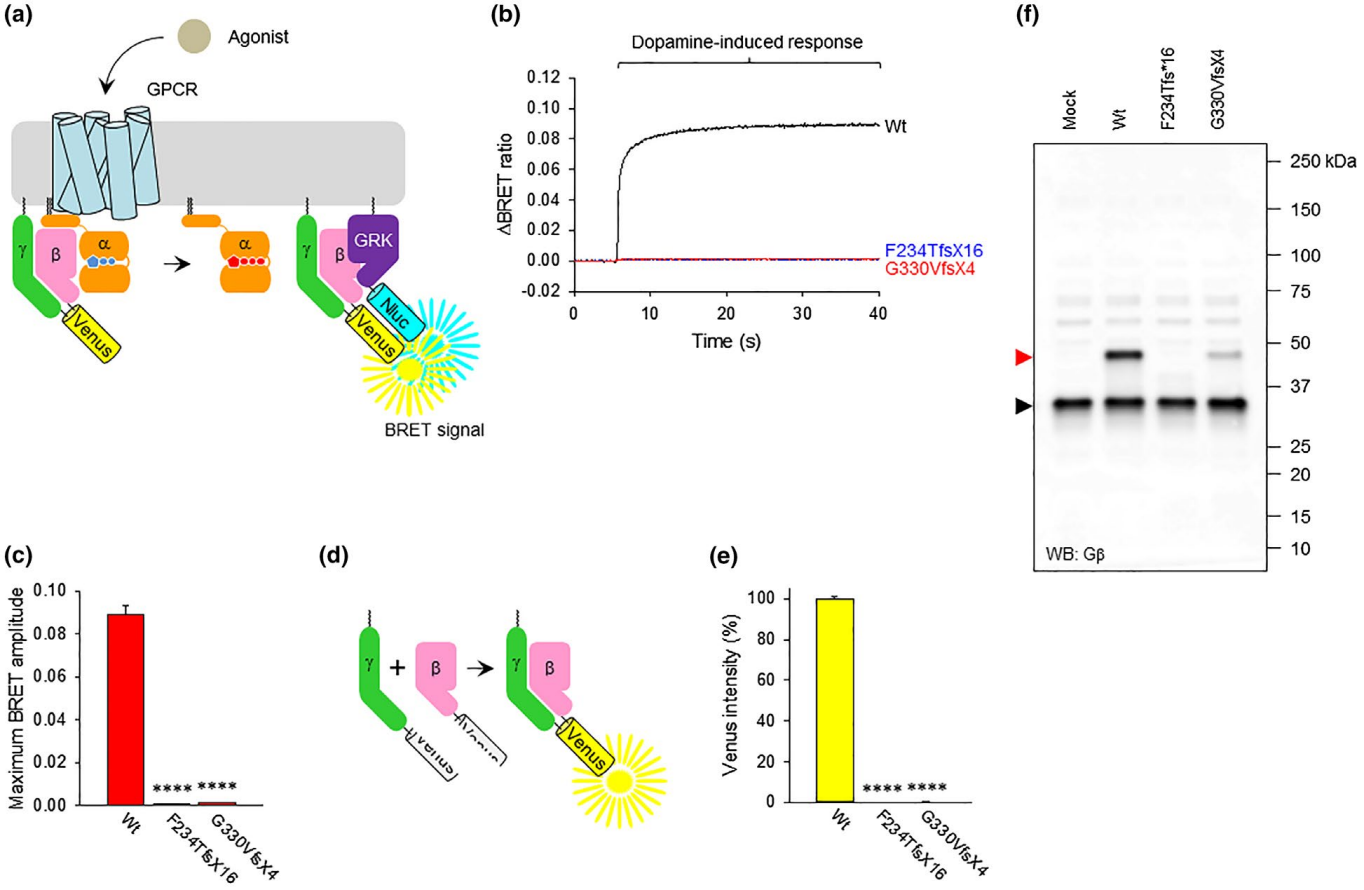
Effect of Gβ1 mutations on its function. (a) BRET assay design for monitoring G protein activation. Dopamine stimulation of cells transiently transfected with D2R, GαoA with BRET sensors results in the dissociation of Venus‐Gβγ from the heterotrimer. Released Venus‐Gβγ dimers become available for interaction with the masGRK3ct‐Nluc‐HA reporter, producing the BRET signal. (b) Real‐time monitoring of G protein activation by the dopamine D2 receptor (D2R). HEK293 T/17 cells were transfected with D2R, GαoA, Venus 1‐155‐Gγ2, and masGRK3ct‐Nluc‐HA, together with Venus 156‐239‐Gβ1 (wild type or mutant). Dopamine at 100 µM concentration was applied at time 5 seconds and the BRET signal was followed across time. (c) Functional assessment of two Gβ variants to mediate agonist‐induced signaling. The values of agonist‐induced maximum BRET amplitude were plotted as a bar graph. Mean ± SEM from three independent experiments were shown. (d) Schematic presentation of the BiFC assay to test Gβγ dimer formation. Two non‐fluorescent fragments of Venus fused to Gβ (Venus 156‐239‐Gβ1) and Gγ (Venus 1‐155‐Gγ2) are brought together by interactions between Gβ and Gγ and produces a yellow fluorescent protein, Venus. (e) Quantitative assessment of Gβγ dimer formation of Gβ1 mutants. Venus intensity of transiently transfected HEK293 T/17 cells to perform BRET assay was measured, and mean ± SEM from three independent experiments was plotted as a bar graph. (f) Expression level of Gβ1 mutants. Western blotting (WB) was performed with anti‐Gβ antibody to detect Venus 156‐239‐Gβ1 proteins. The bands of Venus 156‐239‐Gβ1 and endogenous Gβ are indicated by red and black arrowheads, respectively. Mock refers to cells transfected with an empty vector (pcDNA3.1(+)). Statistics: One‐way ANOVA followed by Dunett's post hoc test was conducted with GraphPad Prism Ver. 6 (****p < 0.0001). *GNB1* GenBank reference sequence NM_002074.4.

In order to confirm that the variants identified in these two patients are in fact LoF, we first examined the functionality of these mutants by performing the experiments for GPCR‐induced G protein activation. For this experiment, we chose the well‐established Bioluminescence Resonance Energy Transfer‐based (BRET) assay (Lohmann et al., 2017; Masuho, Ostrovskaya, et al., 2015). In this assay, agonist stimulation of GPCR induces the dissociation of Gα and Venus‐tagged Gβγ and causes the interaction of Venus‐Gβγ and its binding partner, masGRK3ct‐Nluc‐HA sensor, consequently increasing BRET signal (Figure 3a). In order to model the splice variant p.Phe234Thrfs*16, we generated a cDNA sequence that excluded the 38 base pairs at the start of exon 10 that are skipped by alternate splicing. We modeled the truncating variant p.Gly330Valfs*4 by excluding the two deleted base pairs from the cDNA sequence. These Gβ variants were transfected individually into HEK293T/17 cells with dopamine D2 receptor (D2R), Gαo, Gγ2, and masGRK3ct‐Nluc‐HA to monitor D2R‐Go signal transduction. As expected, the application of dopamine activates Go and increased BRET signal when wild‐type Gβ1 was used (Figure 3b,c). However, we did not see dopamine‐induced response with the cDNA constructs modeling the patient variants. Next, we assessed ability of these variants to form constitutive dimers with the Gγ subunit by bimolecular fluorescence complementation (BiFC; Figure 3d; Hu & Kerppola, 2003). Although wild‐type Gβ1 formed a functional dimer with Gγ2 as evidenced by Venus fluorescence, no such signal was detected from the two variants (Figure 3e). Direct analysis of Gβ1 expression at the protein level by Western blotting (Figure 3f) revealed no detectable protein from the p.Pher234Thrfs*16 construct and severely reduced levels of protein from the p.Gly330Valfs*4 construct. These results indicate that both p.Phe234Thrfs*16 and p.Gly330Valfs*4 are complete LoF variants. We hypothesize that the patient variants result in protein products that are completely non‐functional likely due to instability resulting from the missing portions of the protein sequence.

## 4 DISCUSSION

Here, we present two additional patients with a reported likely pathogenic variant and a variant of uncertain significance in *GNB1,* and phenotypes including developmental delay, intellectual disability, hypotonia, and psychiatric symptoms. Unlike the majority of previously reported patients, both patients have non‐missense variants. We performed functional testing on the presumed LoF variants using BRET‐based assays to examine GPCR‐induced G protein activation and BiFC experiments to assess Gβ1/Gγ subunit interaction. These results showed the proteins modeling patient variants lack normal G protein activation and interaction with the Gγ subunit. As such, these likely represent complete LoF variants. These cases add further evidence to the argument that haploinsufficiency of *GNB1* is a mechanism leading to neurodevelopmental disease in humans. Additional evidence supporting that haploinsufficiency of *GNB1* is damaging is the fact that the gene is highly intolerant to both missense and LoF variation, as well as the fact that there are no deletions encompassing the entire gene listed in the Database of Genomic Variants (MacDonald, Ziman, Yuen, Feuk, & Scherer, 2014) which represents copy number variation found in the normal population. Conversely, there are 63 deletions encompassing the gene listed as pathogenic in the Decipher database (Firth et al., 2009) of affected individuals. However, while recent pre‐print studies of heterozygous mice modeling the missense K78R variant (Colombo et al, 2019, https://doi.org/10.1101/697235, pre‐print) do seem to recapitulate human phenotypes, heterozygous knockout mice do not seem to display a disease phenotype (Okae & Iwakura, 2010). Therefore, additional examples of haploinsufficiency in humans associated with relevant phenotypes are needed as well as protein quantitation in patient samples to concretely associate haploinsufficiency in *GNB1* with neurodevelopmental disorder.

Previously there have only been four reported individuals with presumed LoF variants in *GNB1* reported by Lohmann et al. Two of the individuals had splice variants; a c.268‐1G>T variant predicted to ablate the splice acceptor site upstream of exon 7, and a c.917‐1G>T variant predicted to disrupt the splice acceptor site upstream of the penultimate exon 11. Additionally, two individuals were reported with truncating variants; a c.272_275del; p.His91Profs*10 deletion predicted to cause a transcript truncated at exon 7 which would most likely undergo NMD, as well as a c.915_916del; p.Gly306Cysfs*6 deletion which is not predicted to cause NMD but would result in a protein product missing the terminal 29 amino acids of the protein. The reported phenotypes observed in these patients include global developmental delay, hypotonia, brain abnormalities, ophthalmological abnormalities, epilepsy, and growth delay. However, the severity to which these individuals were affected (i.e., if independent walking and speech were obtained) is not clear.

The phenotypes of the patients reported here, while consistent with previous reports, do appear to be milder in severity. Interestingly, both patients were originally suspected to have Prader‐Willi or Fragile X syndromes based on their developmental delays, hypotonia, psychiatric symptoms, and obesity. While we do not know if obesity was present in the patients reported in Lohmann et al with LoF variants, this phenotype may represent a hallmark of the spectrum associated with haploinsufficiency in *GNB1*. The milder phenotype observed in these cases may be due to a wide spectrum of expressivity associated with damaging variants in *GNB1*. For instance, Petrovski et al. reported a patient with a p.Gly77Ser missense variant who was severely affected with severe global developmental delay, brain abnormalities, and epilepsy without the ability to ambulate independently. However, they also reported a patient with a nearby p.Lys78Arg missense variant who displayed a considerably milder phenotype including mild intellectual disability with the ability to walk and speak. It is also possible that the milder phenotypes are due to the fact that haploinsufficiency, or an overall decrease in the amount of functional protein, is less damaging than the presence of a defective protein that interferes with normal G‐protein complex formation and function.

In summary, we present two patients with novel LoF variants in *GNB1* and neurodevelopmental phenotypes including intellectual disability, hypotonia, obesity, as well as seizures, and psychiatric symptoms in one patient. Functional analysis of these variants demonstrated that the encoded proteins are unable to induce normal G protein activation and are thus LoF variants. These patients help support the evidence that haploinsufficiency in *GNB1* is a disease‐causing mechanism and can help in the future interpretation and classification of LoF variants in this gene.

## CONFLICT OF INTEREST

No conflicts of interest for any of the authors.

## AUTHORS’ CONTRIBUTIONS

LSR, IM, FPV, KAM, EWK designed and organized the study and wrote the paper that was reviewed and edited by all the authors. IM, CTS, TLS, KJC, KAM designed and performed the experimental assays. LG, PNP, KW cared for the patients and acquired the clinical data. All authors read and approved the manuscript.

## Data Availability

Genomic and RNA sequence data cannot be deposited in public databases due to restrictions imposed by the IRB. The data that support the findings of this study are available from the corresponding author upon reasonable request.
